# Establishing research priorities for patient safety in emergency medicine: a multidisciplinary consensus panel

**DOI:** 10.1186/s12245-014-0049-9

**Published:** 2015-01-23

**Authors:** Amy C Plint, Antonia S Stang, Lisa A Calder, for the Priorities in Patient Safety Research in Emergency Medicine Consensus Panel

**Affiliations:** Department of Pediatrics, University of Ottawa, 401 Smyth Road, Ottawa, Ontario K1Y 4E9 Canada; Department of Emergency Medicine, University of Ottawa, The Ottawa Hospital - Civic Campus, 1053 Carling Ave, Ottawa, Ontario K1Y 4E9 Canada; The Children’s Hospital of Eastern Ontario, 401 Smyth Road, Ottawa, Ontario K1H 8 L1 Canada; Department of Pediatrics, University of Calgary, 2888 Shaganappi Trail NW, Calgary, Alberta T3B 4Z6 Canada; Department of Community Health Sciences, University of Calgary, 3280 Hospital Drive NW, Calgary, Alberta T2N 4Z6 Canada; The Alberta Children’s Hospital, 2888 Shaganappi Trail NW, Calgary, Alberta T3B 6A8 Canada; Clinical Epidemiology Program, Ottawa Hospital Research Institute, The Ottawa Hospital - Civic Campus, 1053 Carling Ave, Ottawa, Ontario K1Y 4E9 Canada

**Keywords:** Emergency medicine, Patient safety

## Abstract

**Background:**

Patient safety in the context of emergency medicine is a relatively new field of study. To date, no broad research agenda for patient safety in emergency medicine has been established. The objective of this study was to establish patient safety-related research priorities for emergency medicine. These priorities would provide a foundation for high-quality research, important direction to both researchers and health-care funders, and an essential step in improving health-care safety and patient outcomes in the high-risk emergency department (ED) setting.

**Methods:**

A four-phase consensus procedure with a multidisciplinary expert panel was organized to identify, assess, and agree on research priorities for patient safety in emergency medicine. The 19-member panel consisted of clinicians, administrators, and researchers from adult and pediatric emergency medicine, patient safety, pharmacy, and mental health; as well as representatives from patient safety organizations. In phase 1, we developed an initial list of potential research priorities by electronically surveying a purposeful and convenience sample of patient safety experts, ED clinicians, administrators, and researchers from across North America using contact lists from multiple organizations. We used simple content analysis to remove duplication and categorize the research priorities identified by survey respondents. Our expert panel reached consensus on a final list of research priorities through an in-person meeting (phase 3) and two rounds of a modified Delphi process (phases 2 and 4).

**Results:**

After phases 1 and 2, 66 unique research priorities were identified for expert panel review. At the end of phase 4, consensus was reached for 15 research priorities. These priorities represent four themes: (1) methods to identify patient safety issues (five priorities), (2) understanding human and environmental factors related to patient safety (four priorities), (3) the patient perspective (one priority), and (4) interventions for improving patient safety (five priorities).

**Conclusion:**

This study established expert, consensus-based research priorities for patient safety in emergency medicine. This framework could be used by researchers and health-care funders and represents an essential guiding step towards enhancing quality of care and patient safety in the ED.

**Electronic supplementary material:**

The online version of this article (doi:10.1186/s12245-014-0049-9) contains supplementary material, which is available to authorized users.

## Background

Patient safety is a health-care priority. While the emergency department (ED) is considered a high-risk setting for safety events [1,2], patient safety in the context of emergency medicine is a relatively new field and one in need of further study. Available evidence demonstrates that the ED is a source of patient safety events for patients admitted to hospital [2,3] and that events are common among patients discharged from the ED [2,4]. Studies also suggest that the types and causes of patient safety events among patients discharged from the ED differ from admitted patients [2,3,5]. Factors which contribute to the ED as a high-risk setting include high patient volume, patient acuity and complexity, a work environment characterized by time constraints, multiple interruptions and disrupted sleep cycles for health-care workers, high-risk diagnostic and therapeutic interventions, and variable levels of physician training [1]. The need for ED-based patient safety research is made more pressing by increased mortality [6,7] and treatment delays [8] associated with ED crowding and long wait times.

To date, no overarching research agenda for patient safety in emergency medicine has been established. The objective of this study was to establish patient safety-related research priorities for emergency medicine that would provide a foundation for high-quality research, important direction to both researchers and health-care funders, and an essential step in improving health-care safety and outcomes in the ED setting.

## Methods

### Consensus procedure

We conducted a four-phase, multidisciplinary expert consensus procedure. The Research Ethics Board at the Children's Hospital of Eastern Ontario (Ottawa, Ontario, Canada) approved this project.

### Expert panel

We used a multistep approach to identify panel participants with broad representation: clinicians (physicians, nurses, pharmacists, and other health professionals); researchers from patient safety, adult and pediatric emergency medicine, nursing, pharmacy, and mental health; and representatives from patient safety organizations and health-care administration. We identified a range of experts with this representation from the United States (US) and Canada and formally surveyed them explaining the purpose of our consensus process and asking them to identify individuals appropriate for the expert panel. We approached 36 identified experts, invited them to participate in the panel, and using a snowball sampling approach, asked them to suggest other potential panel members. The 19-member panel consisted of clinicians (generalist and pediatric emergency physicians and nurses), administrators and researchers from adult and pediatric emergency medicine, patient safety, pharmacy, and mental health, as well as representatives from patient safety organizations (further expert panelist details are provided in Additional file 1).

### Phase 1: research priority identification

To identify our initial list of research priorities, we surveyed our expert panel for suggestions (*n* = 19). We also used convenience sampling to survey representatives from the Emergency Medicine Patient Safety Foundation and Canadian Patient Safety Institute and patient safety researchers (*n* = 7), directors of all Canadian pediatric academic EDs (*n* = 14), trauma directors from hospitals accredited by the Trauma Association of Canada (*n* = 11), and leaders of three pediatric emergency medicine research networks (*n* = 5). We used purposeful sampling (to represent a wide geographic area) to survey ED directors from Canadian community hospitals (*n* = 35) selected from a research and clinical partnership network (Translating Emergency Knowledge for Kids http://trekk.ca/).

Potential respondents were sent an email invitation to participate. The online survey contained an open-ended question to elicit research priorities pertaining to patient safety research in emergency medicine (see Additional file 2). There was no limit on the number of priorities a respondent could identify. An open-ended question was used as opposed to an existing patient safety taxonomy or classification system to reduce biasing respondent answers. Non-responders received two email reminders.

### Phase 2: first consensus round with expert panel

In the second phase, we used simple content analysis to group identified priorities from the phase 1 survey. A first consensus round was then conducted online with the expert panel to determine their level of agreement on the identified items as patient safety-related research priorities for emergency medicine. We used a nine-point Likert-type scale that ranged from ‘strongly disagree’ to ‘strongly agree’ and determined *a priori* that priorities rated by ≥70% of the panelists as ‘moderately disagree, disagree, or strongly disagree’ would be discarded. At this stage, panelists could also suggest additional priorities that were not among those listed.

### Phase 3: in-person (round two) consensus meeting

In the third phase, we held an in-person meeting of the expert panel in Ottawa, Canada, on 26 and 27 September 2012. Panelists reviewed the anonymized group rating from the first consensus round for each priority alongside their own responses. A facilitated discussion (by AS) was held for each priority, and, if necessary, the priority was re-phrased. The categorization of priorities was also refined during the discussion. Priorities could be eliminated by consensus.

### Phase 4: third consensus round

Following the in-person meeting, panelists indicated their agreement with the remaining priorities via an online survey using the same nine-point Likert-type scale from phase 2. During our in-person meeting, we established that only priorities rated as ‘agree’ or ‘strongly agree’ by ≥70% of respondents in the final consensus round would be retained.

## Results

### Research priorities identification and refinement

In phase 1, 91 individuals were surveyed for suggestions of research priorities with 32 (35.2%) responding. The vast majority of non-respondents were directors of community hospital EDs (5/35 surveyed responding). One hundred and seventeen research priorities were suggested with a mean suggestion of 3.7 (range 1 to 10) per respondent. After removal of duplicates and content analysis, 66 unique research priorities were identified within 7 broad categories: 1) standardized terminology, 2) measurement and reporting, 3) epidemiology, 4) understanding human and environmental factors related to patient safety, 5) patient perspective, 6) impact, and 7) interventions.

### Expert consensus

After three rounds, consensus was reached for 15 patient safety-related research priorities for emergency medicine based on the initial 66 priorities. The final priorities are divided into four categories (Table 1). While not an *a priori* objective of the consensus process, discussion at the in-person meeting resulted in the identification of three important guiding principles for patient safety research in emergency medicine: (1) when conducting research in patient safety, a clear identification and reporting of the taxonomy used is strongly recommended; (2) in order to address these research priorities, we need to build and sustain truly collaborative partnerships between clinicians and safety scientists; and (3) innovative and novel methods beyond conventional clinical investigative techniques are required.

**Table 1:** **Consensus-based priorities for patient safety research in emergency medicine**

	**Consensus-based priorities**
I	Methods to identify patient safety issues
	Developing or evaluating methods to identify and understand adverse events among ED patients
	Developing or evaluating methods identify and understand problems in ED care that lead to subsequent unplanned health-care utilization
	Developing or evaluating methods to identify and understand near misses
	Developing or evaluating methods to identify and understand diagnostic errors in emergency medicine
	Developing or evaluating methods to learn from patient safety events*
II	Understanding human and environmental factors related to patient safety
	Completing foundational work to understand how people work in the challenging, unforgiving environment of the ED (e.g., understanding how individuals working in teams sense problems and formulate plans to resolve them; understanding how individuals working in teams recognize and negotiate goal conflict; and understanding how people adapt to the unexpected (how can we better support their ability to anticipate, monitor, respond, and learn)
	Understanding how system factors (e.g., provider characteristics, technologies, and physical environment, crowding) influence patient safety events in the ED
	Understanding the influence of coordination/transition issues across the continuum of patient care (e.g., handover, transfers between hospitals, and transfers between units) on patient safety events
	Understanding the most important precursor events and unsafe situations that lead to adverse events
III	Patient perspective
	Exploring the role of patients and families in detecting, reporting, and preventing patient safety events*
IV	Interventions to promote patient safety
	Evaluating the impact of feedback and reporting to providers (e.g., patient outcome feedback, performance reviews, M and M rounds) on patient safety events
	Evaluating the impact of simulation on patient safety events*
	Evaluating the impact of cognitive support interventions on patient safety events*
	Developing and evaluating interventions to improve diagnostic accuracy
	Developing and evaluating interventions to address coordination/transition issues across the continuum of patient care (e.g., handover, transfers between hospitals, and transfers between units)

*The term ‘patient safety event’ is used in this context to encompass adverse events, near misses, apparent hazards, and diagnostic errors.

## Discussion

We present the first overarching consensus-based patient safety research priorities developed for emergency medicine. Previous work has suggested a potential disconnect between safety concerns identified by national bodies in charge of patient safety and those identified by emergency medicine physicians [9]. Concerns ranked highly by emergency physicians, but not previously considered in national patient safety initiatives, include the effects of the availability of expert consultation and of follow-up care on patient safety [9]. This is also in keeping with findings that unintended patient safety events in the ED are often related to cooperation with other departments [10]. These concerns were identified by our expert panel and highlight the need to understand the influence of coordination/transition issues across the continuum of patient care on patient safety events.

Research priorities for patient safety specifically within the context of ED crowding have been published [11]. While both this work and ours involved expert, consensus-based recommendations, the scope of priorities identified in our process provides a broader agenda for patient safety research in emergency medicine, beyond ED crowding. Both processes identified the need to develop innovative and novel methods outside the conventional clinical investigative techniques to measure, evaluate, and understand patient safety events. Our priorities align well with research agendas laid out by large national patient safety organizations. The five areas of research focus identified by the National Patient Safety Foundation [12] are reflected in our priorities to develop and evaluate methods to identify and understand patient safety problems, understand human and environmental factors related to patient safety, and evaluate interventions that aim to improve patient safety in the ED. Our priorities are also reflected within the patient safety research agenda set by the Agency for Healthcare Research and Quality which include understanding the epidemiology of medical error and patient safety, issues related to transitions of care, and understanding and evaluating interventions to improve patient safety [13]. Finally, our priority to understand how transitions across the continuum of care is one of the six priority areas for patient safety research identified by the WHO [14]. Our focus on ED-specific research priorities also fits well within the WHO goal of focusing research on identifying locally effective and affordable solutions and local research priorities [14].

As highlighted by our guiding principles, the next step in furthering the patient safety research agenda in the ED setting is for researchers to align with safety scientists to explore novel research methods to address the priorities we identified. Examples of novel research methods that have been identified for application in patient safety research include methods from engineering such as process mapping and probabilistic risk assessment, direct observation using ethnographic approaches, and methods from organizational psychology and sociology to assess organizational culture. Collaboration across disciplines is essential to determine the optimal research method, or methods, to translate this agenda into relevant and feasible research that will improve patient safety in the ED [15].

### Limitations

Although we engaged a wide selection of stakeholders including individuals with considerable experience in patient safety and patient safety research, representatives from patient advocacy groups, large funding agencies, and safety science (such as human factor engineers, complex systems experts, psychologist, and social scientists) were not involved and could have provided additional contributions to the research agenda. Furthermore, although the response rate to our initial survey for suggested priorities was low at 35.2%, when we examined our low response rate, it was primary related to the poor response from community ED directors and we sent multiple responders to non-responders in an attempt to improve response rates. It is difficult to interpret the low response rate from community ED directors, and it may reflect multiple administrative demands on their time. It is unlikely a reflection of their lack of interest in patient safety. It is important to note that we purposefully sampled a wide audience in an attempt to seek input from individuals who were outside the area of patient safety research. Next steps could involve reviewing the identified priorities with safety scientists to gain further insights into important patient safety research topics that may not be typically discussed in the clinical setting.

## Conclusions

Given the high-risk environment of the ED and the evidence of patient safety issues related to ED care, establishing a focused research agenda in patient safety in emergency medicine is both timely and critical. We believe that this consensus-driven process will assist researchers, administrators, and funders in focusing efforts for improving the safety of patients who receive care in the ED.

## Additional files

Additional file 1:
**Expert panel members.** Detailed information regarding expert panel members (names, affiliations, training, and expertise). Additional file 2:
**Initial survey tool.** Initial survey sent to elicit suggested research priorities from a wide range of stakeholders.

## Abbreviations

ED: emergency department
US: United States
