# Supplementary material for: Establishing research priorities for patient safety in emergency medicine: a multidisciplinary consensus panel
Source: Int J Emerg Med. 2015 Jan 23;8:1. doi: 10.1186/s12245-014-0049-9 (PMC4384522; doi:10.1186/s12245-014-0049-9)
Supplement: Additional file 1: — Expert panel members. Detailed information regarding expert panel members (names, affiliations, training, and expertise). [file 12245_2014_49_MOESM1_ESM.doc]

| TEAM MEMBER | POSITION | STUDY ROLE |
| --- | --- | --- |
| Amy Plint, MD, MSc | University of Ottawa Tear 2 Clinical Research Chair in Pediatric Emergency Medicine; Associate Professor, Department of Pediatrics and Emergency Medicine, University of Ottawa; Senior Investigator, Children’s Hospital of Eastern Ontario Research Institute, Ottawa; Chair, Pediatric Emergency Research Canada (PERC) | - Pediatric Emergency Medicine - Multi-centre trials - Randomized controlled trial methodology - Research about the emergency management of acute respiratory illness and injuries - Knowledge synthesis |
| Antonia Stang, MD, MBA, MSc | Assistant Professor, Department of Pediatrics, University of Calgary; PERC Site Representative; Member Alberta Provincial Pediatric Indicator Working Group; Member American Academy of Pediatrics Section of Emergency Medicine Performance Measurement Task Force | - Health services research - Patient safety - Quality improvement - Performance measurement |
| Lisa Calder, MD, MSc | Assistant Professor, Department of Emergency Medicine, University of Ottawa; Associate Scientist, Ottawa Hospital Research Institute. | - Patient safety and adverse event reporting in the ED setting - Adult emergency medicine - Completed two patient safety fellowships |
| Stacy Ackroyd-Stolarz, MSc, PhD | Department of Emergency Medicine, Dalhousie University, Halifax, Nova Scotia, Canada; Patient Safety Scientist, Capital District Health Authority, Halifax, Nova Scotia | - Patient safety research focused on the elderly population in the ED - Epidemiology - Completed postdoctoral fellowship in patient safety |
| Maala Bhatt, MD, MSc | Assistant Professor, Department of Pediatrics, Children’s Hospital of Eastern Ontario* | - Pediatric procedural sedation safety - Adverse event reporting - Pediatric Emergency Medicine |
| Jim Chamberlain, MD | Division Chief, Emergency Medicine, Children’s National Medical Center; Professor of Pediatrics and Emergency Medicine, George Washington University; co-lead Pediatric Emergency Care Applied Research Network (PECARN) Safety Working Group | - Quality assessment research - Safety and quality improvement - Pediatric emergency medicine |
| Karen Cosby, MD | Department of Emergency Medicine, Rush University Medical School and Department of Emergency Medicine, Cook County, Co-Director, Emergency Ultrasound Fellowship Program, Health and Hospital System, Chicago, Illinois | - Patient safety research - Ultrasound in the emergency department - Emergency medicine |
| Ken Farion, MD | Associate Professor, Departments of Pediatrics and Emergency Medicine, University of Ottawa; Medical Director and Chief, Pediatric Emergency Medicine, Children’s Hospital of Eastern Ontario (CHEO), Ottawa, Ontario; Medical Director, Quality and Systems Improvement, CHEO; PERC Site Representative | - Emergency Medicine - Health services delivery in the pediatric emergency department - Quality and patient safety - Data capture using electronic health record - Lean process design and quality improvement |
| Olavo Fernandes, PharmD | Clinical Director of Pharmacy, University Health Network, Toronto, Ontario and Leslie Dan Faculty of Pharmacy, University of Toronto, Toronto, Ontario | - Pharmacy leadership - Patient safety and medication reconciliation - Medication information transfer - Design and implementation of tools for medication reconciliation and information transfer. |
| Abigail M Hain, MSc Nursing | Director of Education, Canadian Patient Safety Institute, Ottawa, Ontario | - Quality and patient safety education - Healthcare education - Critical care |
| Mona Jabbour, MD, MEd | Associate Professor, Department of Pediatrics, University of Ottawa; Vice-Chair, Department of Pediatrics, Children’s Hospital of Eastern Ontario; Co-Chair Pediatric Emergency Administrators for Children’s Hospitals | - Translation of best evidence into practice - Pediatric emergency medicine - Health care administrationPediatric emergency medicine |
| Amanda Newton, RN, PhD | Assistant Associate Professor, Department of Pediatrics, Faculty of Medicine & Dentistry, University of Alberta, Edmonton, Alberta, Canada; Clinician Scientist (Child and Adolescent Psychiatry), Stollery Children’s Hospital, Edmonton | - Children’s emergency mental health services - Mixed method studies - Integrated knowledge translation |
| Adam Oster, MD | Foothills Medical Center, Calgary, Alberta Children’s Hospital and Department of Emergency Medicine, University of Calgary, Calgary, Alberta | - Adult emergency medicine - Quality improvement |
| Richard M Ruddy, MD | Professor of Pediatrics, University of Cincinnati College of Medicine; Director, Division of Emergency Medicine, Cincinnati Children’s Hospital Medical Center; Vice Chair, PECARN Steering Committee; Member PECARN Safety and Quality Working Group | - Use of Improvement Science in health care - Building of more reliable systems through decision support - Health care administration; - Pediatric emergency medicine |
| Kathy Shaw, MD, MSCE | Associate Chair and Patient Safety Officer, Department of Pediatrics; Nicholas Crognale Endowed Chair and Chief, Division of Emergency Medicine; Professor of Pediatrics at Children’s Hospital of Philadelphia, Perelman School of Medicine, University of Pennsylvanian; co-lead PECARN Safety Working Group; Chair of the Committee of Pediatric Emergency Medicine for the American Academy of Pediatrics | - Quality improvement initiatives and patient safety in emergency medicine - Diagnosis and management of acute illness and injury - Pediatric emergency medicine |
| Douglas Sinclair, MD | Executive Vice President and Chief Medical Officer, St Michael’s Hospital, Toronto, Ontario; Professor, Department of Medicine, University of Toronto, Toronto, Ontario | - Patient safety - Medical error - Health care administration - Pediatric and adult emergency medicine |
| Kim Stelmacovich, BScOT, MHSc | Senior Director, Canadian Patient Safety Institute, Ottawa, Ontario; Adjunct Faculty, University of Toronto Institute for Health Policy, Toronto, Ontario | - Implementation of patient safety programs - Operationalizing strategic priorities - Quality improvement |
| James Stempien, MDBSC MD CCFP EM FCFP CCPE | Head, Department of Emergency, Saskatoon Health Region, Saskatoon, Saskatchewan; Clinical Assistant Professor, University of Saskatchewan | - Emergency medicine - Health care administration - LEAN Leader Training Certificate - Expertise and experience in emergency department flow and LEAN initiatives - Consultant in Government of Saskatchewan “No ER Waits by 2017” initiative - Quality improvement research and initiatives |
| Robert Wears, MD, MSc, PhD | Professor, Department of Emergency Medicine, University of Florida, Jacksonville, Florida and Imperial College London, London, UK | - Patient safety - Industrial safety - Emergency medicine |

* At time of consensus conference, Maala Bhatt was a member of the Department of Pediatrics, McGill University, Montreal, Quebec
