# Supplementary material for: Establishing research priorities for patient safety in emergency medicine: a multidisciplinary consensus panel
Source: Int J Emerg Med. 2015 Jan 23;8:1. doi: 10.1186/s12245-014-0049-9 (PMC4384522; doi:10.1186/s12245-014-0049-9)
Supplement: Additional file 2: — Initial survey tool. Initial survey sent to elicit suggested research priorities from a wide range of stakeholders. [file 12245_2014_49_MOESM2_ESM.docx]

**Appendix file 2: Initial Survey Tool**

Establishing priorities for patient safety research in the emergency department setting

Please complete the survey below.

Thank you!

What areas of patient safety research in the Emergency Department setting do you see as a priority? Please list specific research questions and reasons why you see this as a priority if possible and briefly explain why you see this as a priority. We are interested in priorities for both adult and pediatric patients.

Examples of possible research priorities could include:

Epidemiology:

1. How common are adverse events in the Emergency Department?
2. How many adverse events are preventable in the Emergency Department?
3. What are risk factors for adverse events?

Measurement:

1. Development of specific tools to identify adverse events within the Emergency Department.
2. Development of measurement tools for adverse events using electronic health records.

Interventions:

1. Prospective examination of the effect of interventions on occurrence of adverse events in the Emergency Department.

Please list as many priorities as you want

Priority/Question 1 ___________________________

Explain why this is a priority

Priority/Question 2 ___________________________

Explain why this is a priority

Priority/Question 3 ___________________________

Explain why this is a priority

Priority/Question 4 ___________________________

Explain why this is a priority

Priority/Question 5 ___________________________

Explain why this is a priority

Do you want the option to list more priorities?  Yes

 No

Priority/Question 6 ___________________________

Explain why this is a priority

Priority/Question 7 ___________________________

Explain why this is a priority

Priority/Question 8 ___________________________

Explain why this is a priority

Priority/Question 9 ___________________________

Explain why this is a priority

Priority/Question 10 ___________________________

Explain why this is a priority

Do you want the option to list more priorities?  Yes

 No
